# Measuring Well-Being of Migrant Gig Workers: Exampled as Hangzhou City in China

**DOI:** 10.3390/bs12100365

**Published:** 2022-09-27

**Authors:** Tinggui Chen, Weijin Song, Junying Song, Yixuan Ren, Yuzhu Dong, Jianjun Yang, Shuyuan Zhang

**Affiliations:** 1 School of Statistics and Mathematics, Zhejiang Gongshang University, Hangzhou 310018, China; 2 Collaborative Innovation Center of Statistical Data Engineering Technology & Application, Zhejiang Gongshang University, Hangzhou 310018, China; 3 Department of Computer Science and Information Systems, University of North Georgia, Oakwood, GA 30566, USA; 4 Department of Human Resources, Zhejiang University of Water Resources and Electric Power, Hangzhou 310018, China

**Keywords:** gig economy, migrant, well-being, difference analysis of demographic variables, structural equation model

## Abstract

The consistent innovations and applications of information technology drive the vigorous development of the gig economy, and generate gig workers such as food delivery workers and couriers, and make a great contribution to stabilizing employment and increasing income. Gig workers, mostly made up of migrants, and suffer from job and status difficulties. Research on the well-being of migrant gig workers can reveal the practical problems and provide suggestions for narrowing the wealth gap to promote social fairness and justice. Taking Hangzhou city in China as an example, this paper explores the well-being of food delivery workers, couriers, and online car-hailing drivers as representatives of migrant gig workers. Firstly, the relevant data are acquired through the questionnaire. Secondly, the characteristics of this group are analyzed through descriptive analysis, namely: most of them are migrant workers aged between 20 and 39 with low occupation satisfaction due to insufficient social security coverage and limited well-being, despite relatively high income. Based on the analysis of differences in demographic variables and structural equation modeling, the factors affecting the well-being of migrant gig workers are studied, which mainly are occupation satisfaction, social interaction, and social security. The results show that occupation satisfaction is positively affected by family characteristics, social interaction, and social security. In addition, family characteristics and social security positively impact social interaction, but the former has no significant effect on well-being. Finally, this paper enriches the research on the well-being of specific migrant gig workers and gives policy suggestions for enhancing the well-being of migrant gig workers in Hangzhou city from the perspective of optimizing the mechanism, pilot construction, and platform provision.

## 1. Introduction

The accelerating development of the Internet economy and digital technology witnesses the rapid growth of the gig economy. More than a third of the U.S. workforce is in the gig economy, according to Gallup data cited by EMBROKER, an online insurance platform. In addition, relevant forecasts show that in Europe, half of residents aged 15–24 have joined the gig economy; in China, about 400 million people are expected to participate in the gig economy in 2036.Lata et al. [1] noted that gig workers were mostly migrants. In the Chinese context, they usually referred to domestic migrants. They are mainly engaged in catering, express delivery, transportation, and other service industries. On the one hand, the gig economy can increase employment opportunities; it can also expand the size of the middle-income group, thereby narrowing the wealth gap and promoting social fairness and justice. On the other hand, however, there are serious defects in terms of working environment, protection of rights and interests, and social integration, which take a heavy toll on safeguarding migrant gig workers’ equal rights, achieving rapid economic development, and promoting social harmony and stability. Based on this, analyzing the living dilemma encountered by this group and proposing countermeasures are pivotal to realizing the healthy development of the labor market. Generally speaking, the well-being can measure the individual’s satisfaction with the status quo of life, thereby revealing the individual’s predicament. The relief of the predicament depends on analyzing the factors affecting well-being. Therefore, this paper analyzes the living conditions of migrant gig workers from the perspective of well-being and further provides suggestions for improving well-being according to the influencing factors, to promote the healthy development of the labor market and realize social harmony and stability.

At present, there are few studies on the well-being of migrant gig workers, which have limited research objects and research contents. With regard to research objects, most studies focus on analyzing the well-being of certain specific groups, such as food delivery workers and couriers, while ignoring the similarities in some migrant gig jobs. For example, by exploring the living conditions of couriers and their integration into the city, Fang et al. [2] found that they had problems such as work–family imbalance, poor awareness and ability of legal rights protection, and lacked of a sense of belonging. Apouey et al. [3] analyzed an online survey and found that the psychological pressure of food delivery workers was significantly reduced during the lockdown caused by COVID-19. Benediktet al. [4] investigated the work situation and well-being of Uber drivers and found that work flexibility could significantly affect well-being. With regard to research content, most studies focus on analyzing the cognition and interpretation, labor affiliation, and rights protection of migrant gig workers, ignoring the well-being status of this group and its influencing factors, and thus lacking in-depth analysis from the perspective of individual feelings. In addition, through empirical surveys conducted in Beijing and Chengdu, Chen et al. [5] found that the development of the digital economy had led to the frequent occurrence of unstable employment manners, resulting in the shortage of social and labor rights of relevant practitioners. Based on the boundaryless career theory, Duggan et al. [6] found that the platform algorithm control under the gig economy limited the career development of practitioners through in-depth interviews.

In fact, the gig economy mainly consists of two types: one is the traditional way of working with the help of platforms to match work projects, which is mainly focused on service industries such as express delivery, transportation, equipment maintenance and catering, and the other is a new manner of working based on platforms, such as Internet marketing specialists. Therefore, this paper focuses on the workers participating in the first gig economy. They have great similarities in terms of personnel composition, labor nature, etc., and researching their well-being as a group can make up for the gaps in the above investigations. In addition, Choudhary and Shiresh [7] pointed out that the life quality of gig-drivers was poor, because of long working hours and fierce peer competition, but their self-perception was good. Based on this, this paper explores well-being and its influencing factors for the migrant gig workers represented by food delivery workers, couriers, and online car-hailing drivers in Hangzhou. On the one hand, it can enrich the well-being research of specific groups, and find the influencing factors of well-being and their internal relations. On the other hand, it is beneficial for the government, enterprises, and other units to actively introduce various policies to improve the well-being of such groups and provide suggestions for narrowing the wealth gap and advancing social fairness and justice.

The structure of the paper is arranged as follows: Section 2 discusses the literature review and hypotheses development. Section 3 presents the research framework of this paper. Section 4 describes the research methods. Section 5 presents the results. Section 6 includes the discussions, suggestions and limitations, and Section 7 presents the conclusions.

## 2. Literature Review and Hypotheses Development

The gig economy in the Internet era has aroused the attention of many organizations and institutions, with a focus on the opportunities and challenges presented by the gig economy. Migrant gig workers who are engaged in different jobs but have certain similarities and representativeness can be explored as a whole, which can make a more comprehensive analysis of their well-being. Generally speaking, the characteristics of the group, social relations, and work situation will have a degree of influence on well-being. For the government, grasping the influencing factors of well-being is conducive to social harmony and stability, as well as the vigorous development of the digital economy. This paper analyzes the literature and proposes hypotheses from the following two aspects: (1) the concept of the gig economy and the living conditions of migrant gig workers; (2) the evaluation of well-being, and the analysis of influencing factors.

(1) The Concept of Gig Economy and Living Conditions of Migrant Gig Workers

Scholars have not yet reached a consensus on the concept of the gig economy. Based on the characteristics of gig work, Tan et al. [8] refers to the gig economy as markets in short-term, on-demand, and typically task-based labor. Through the comparison of the nature of the platform, the nature of workers, and the nature and the scope of the work, Koutsimpogiorgos et al. [9] believed that the gig economy could be narrowly defined as ex ante specified, paid tasks carried out by independent contractors mediated by online platforms. This paper argues that the gig economy is a new labor model with relatively flexible working hours, locations, and methods. In this mode, independent workers engage in on-demand project-based work with the help of the online platform and get corresponding remuneration.

This paper refers to Wang and Zhang’s [10] definition of migrant gig workers, in that they are migrant workers who become “gig economy” practitioners and use it as their only source of income. They are usually engaged in traditional work with the help of online platforms to match work items. Abkhezr and McMahon [11] thought the main reason for migrant workers to participate in the gig economy was that their career development was facing challenges after migration, and the gig economy provided them with opportunities to alleviate their economic problems quickly. However, migrant gig workers still face serious challenges. These challenges are created by a combination of migration status and work situations. For example, after studying migrant food delivery workers in Norway and Switzerland, Newlands [12] found that gig work could not bring them a sense of pride, integration, or family support. Moreover, gig work was temporary and could not provide opportunities for future. Doorn et al. [13] believed that migrant gig workers were vulnerable because they were not only excluded from the local welfare system, but also suffered from health and safety problems brought about by their work. Numerous studies argue that gig workers are suffering from labor exploitation and point to platforms’ algorithmic control of gig workers. The inherent instability of gig work and the misclassification of gig identities are deep-seated reasons for the plight of gig workers. Ashford et al. [14] found that the challenges of gig workers included lack of income security, high mental stress, limited career development, job instability, and little social interaction.

From the above analysis, the existing literature has carried out a relatively comprehensive elaboration on the life plight of migrant gig workers. However, few literatures have studied migrant gig workers with some similarity as a group, which leads to the omission of some common problems. Specific migrant gig workers face challenges in both work and life. Therefore, it is urgent to explore their living situation from the perspective of self-evaluation, and analyze the common problems they face and the influencing factors behind them, so as to improve their current situation.

(2) The Evaluation of Well-being and the Analysis of Influencing Factors

The concept used in this paper belongs to the category of subjective well-being. Represented by Diener [15], he believed that well-being was a comprehensive evaluation of the quality of life made by people based on their own evaluation criteria.

At present, there are few well-being studies directly aimed at migrant gig workers or a single population in this group. To this end, this paper selects keywords such as migrant workers to collect relevant literature on well-being evaluation. In addition, from the perspective of data sources, most of the data in the study come from official survey agencies, and a small part come from questionnaires conducted by researchers. Data for the former are usually more readily available, but may only consider a comprehensive question about well-being, such as data from the Chinese General Social Survey (CGSS). The latter mainly draws on domestic and foreign mature scales to measure well-being in a multi-dimensional and multi-item way. For instance, Medvedev and Landhuis [16] confirmed that the Positive and Negative Affect Scale(PANAS), the Short-form Version of the World Health Organization Quality of Life Measurement Tools (WHOQOL-BREF), the Satisfaction with Life Scale (SWLS), and the Oxford Well-being Questionnaire(OHQ)could be used to assess well-being, and the results were consistent. Xu et al. [17] used the widely used well-being scale in China to measure the well-being of rural migrant workers, namely the Subjective Well-being Scale for Chinese Citizen (SWBS-CC20) and the Multiple Well-being Questionnaire (MHQ). They also found that the reliability of the two scales was good, but the validity was not ideal, so it could not be effectively measured. Taking Zhejiang Province as an example, Xing et al. [18] confirmed that MHQ had good reliability and validity in assessing the well-being of migrant workers, and that the well-being of new-generation migrant workers (who were born after 1980 and gradually moved to cities in the late 1990s) was significantly higher than that of first-generation migrant workers (who were born before 1980 and migrated from the countryside to cities after the reform and opening up). Lee and Zhao [19] believed that the General Health Questionnaire (GHQ) had broad applicability in assessing well-being. In addition, married and healthy individuals with high family income had higher well-being. Liu et al. [20] believed that SWLS could effectively assess the well-being of migrant workers, and found that the absolute economic status of migrant workers was negatively correlated with well-being. By combining SWLS and PANAS to measure well-being, Liu et al. [21] found that the well-being of migrants was low.

In fact, well-being is affected by various factors. Wang and Vander [22] proposed that well-being was related to gender, income, marriage and family, interpersonal relationships, etc.; interestingly, the sense of relative deprivation (someone’s relative deprivation aroused from comparing with his/her past self or the ordinary people around him/her in a certain way and finding himself/herself at a disadvantage) perceived by the individual was the most important factor affecting well-being. Fang and Sakellariou [23] used unconditional quantile regression to find that income, working hours, and health were more likely to affect unhappy migrant workers, while education and social security (such as medical insurance and endowment insurance) were positively related to their well-being. Wu et al. [24] used the questionnaire data to construct a structural equation model, and found that both occupation satisfaction and social support could affect the well-being of food delivery workers. Specifically, job meaning perception and job autonomy affected their occupation satisfaction, and online communication among family members and colleagues affected their social support.

Based on the research results of the above literature, this paper will explore the relationships among family characteristics, occupation satisfaction, social security, social interaction, and well-being.

In this paper, family characteristics are defined as the satisfaction of family life and the degree of harmony in family relations. A warm and harmonious family atmosphere can have an impact on individual well-being. On the one hand, Zhu et al. [25] found that migrant workers who go out to work alone had lower well-being and greater social interaction barriers than migrant workers who migrate with families. On the other hand, a good family life and relationship can provide support for individuals, relieve the negative emotions or pressures brought by work, and then reduce the negative impact of work stress on job satisfaction. This paper thus proposes the following research hypotheses:

**H1a** **:** *Family characteristics positively impact well-being*.

**H1b** **:** *Family characteristics positively impact social interaction*.

**H1c** **:** *Family characteristics positively impact occupation satisfaction*.

Occupation satisfaction generally refers to an individual’s evaluation of his or her work or work experience. In the context of the gig economy, occupation satisfaction mainly includes the degree of satisfaction with job flexibility, job content, compensation, platforms or companies served, and relationships with colleagues. Factors such as working hours, income, and job cognition are all related to job satisfaction and have a significant impact on well-being. Hong et al. [26] found that long working hours was associated with occupational stress, well-being, and depression. This paper thus proposes the following research hypothesis:

**H2** **:** *Occupation satisfaction positively impacts well-being*.

Social security includes a series of social welfare security such as housing security and social insurance, which can provide protection for individuals. Leng and Zhu [27] believed that housing security, such as public rental housing policies and housing price controls, had a significant positive correlation with residents’ well-being. This correlation was more significant for smaller living areas per capita or lower incomes. Under the protection provided by social security, the consumption power, health level, and sense of fairness of individuals are often promoted, which further enhances the sense of well-being. In addition, individuals who have social insurance or participate in many types of insurance are happier, and social insurance is usually paid by organizations. For gig workers, more risks will be encountered in the process of work execution, such as traffic accidents. Social insurance can provide security for work, and the level of security perceived by individuals can affect work emotions. This paper thus proposes the following research hypotheses:

**H3a** **:** *Social security positively impacts well-being*.

**H3b** **:** *Social security positively impacts occupation satisfaction*.

Social interaction is the embodiment of interpersonal relationship and one of the sources of social support. Shao [28] proposed that social support formed by interpersonal relationships (such as friends, colleagues, neighbors, etc.) was the most important factor affecting the well-being of migrant workers. This paper thus proposes the following research hypothesis:

**H4** **:** *Social interaction positively impacts well-being*.

**Figure 1:**
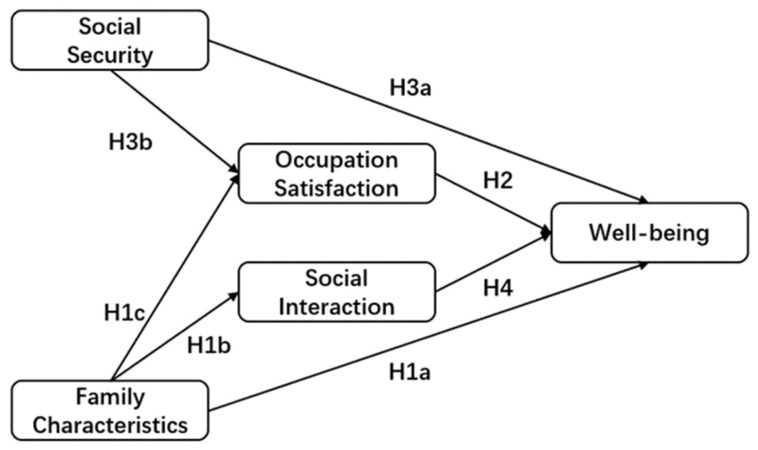
Theoretical Model.

To sum up, the current research on migrant gig workers is insufficient. The existing literature either focuses on the workers as a whole or only focuses on certain occupations, ignoring the similarities and differences among certain occupational categories in the group. Moreover, the existing literature is usually based on occupational predicaments and the protection of rights and interests without considering workers’ evaluation of their own life, which cannot demonstrate their real life and work conditions. Domestic and foreign scholars have fully studied well-being, including various measurement methods of well-being and the analysis of influencing factors. In the measurement of well-being, the main deficiency is that ignoring the particularity of the group will affect the accuracy of the research results. To this end, this paper proposes the following research questions: 1. What is the basic living situation of migrant gig workers? 2. What is the status of the well-being of migrant gig workers? 3. What factors influence their well-being? 4. What factors have the greatest impact on well-being? 5. Is there any relationship among the influencing factors? To answer the above questions, taking food delivery workers, couriers, and online car-hailing drivers as examples, this paper obtains the basic status, well-being, and influencing factors of this group through a questionnaire survey. In addition, the maturity scale selected in this paper is applicable in specific regions and groups. Specifically, for question 1, descriptive statistical analysis and multiple response analysis are used; for question 2, demographic variable analysis is used; for questions 3–5, structural equation model is used; and the proposed theoretical model is shown in Figure 1.

## 3. Research Framework

With the rapid development of the gig economy, the scale of migrant gig workers represented by food delivery workers, couriers, and online car-hailing drivers has expanded rapidly in recent years. At the same time, workers engaged in such industries face prominent problems in terms of the working environment, protection of rights and interests, and policy support. Therefore, it is urgent to explore the current status and influencing factors of migrant gig workers’ well-being, to reveal the real problems encountered by this group in the gig economy environment. Our research outcomes can confirm the results of extant literature in the field of specific migrant gig workers. Additionally, this study can provide suggestions to the government and enterprises from the perspective of enhancing well-being to advance the construction and development of the gig economy, narrow the wealth gap, and promote social fairness and justice.

**Figure 2:**
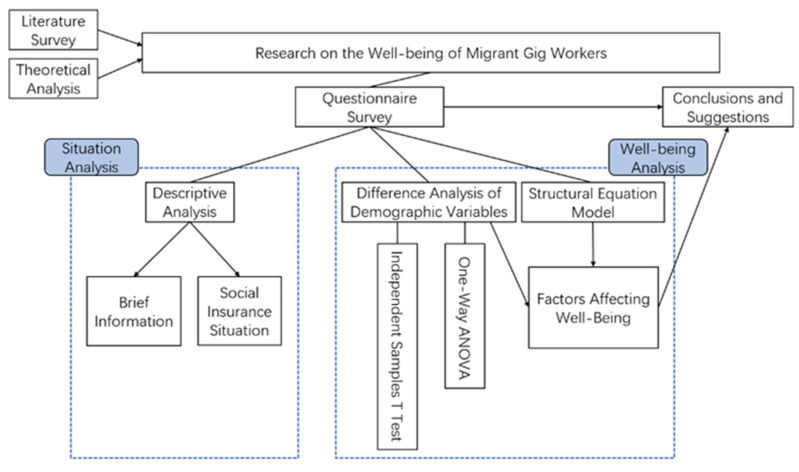
Research Framework.

This paper takes food delivery workers, couriers, and online car-hailing drivers as representatives of the migrant gig workers and the objects of studies. Firstly, the data on their life status and well-being status are obtained through a questionnaire survey. Secondly, descriptive analysis is carried out to acquire information on the basic life status of the group. Thirdly, the difference analysis of demographic variables is conducted, including the independent samples *t*-test and one-way ANOVA. At the same time, a structural equation model (SEM) is constructed to summarize the factors affecting the group’s well-being. Finally, this paper summarizes the above analysis results and puts forward corresponding suggestions. The research framework of this paper is shown in Figure 2.

## 4. Research Methods

This section introduces the research methods, including the determination of the initial survey method and the collection of the final questionnaire data, demonstrating the whole process from program design to data quality assessment. Specifically, this paper focused on questionnaire surveys, taking food delivery workers, couriers, and online car-hailing drivers in Hangzhou city as the survey objects. Firstly, the required samples were obtained by a multi-stage sampling method, and then the questionnaires based on the maturity scale were adopted to obtain respondents information. The data quality is strictly controlled during the implementation of the survey, and the quantity and quality of the questionnaires collected were finally evaluated.

### 4.1. Survey Method

This paper is mainly based on a questionnaire survey, supplemented by a literature survey and field interviews.

A large amount of literature was studied, including but not limited to academic literature, policy documents, news reports, economic development reports, etc. The research on relevant literature guided the investigation and analysis process in this paper and supplemented the further understanding of the research question.

The field interviews were conducted during the pre-investigation stage. The interviewees were the same as the survey respondents, including food delivery workers, couriers, and online car-hailing drivers in Hangzhou. The contents included the basic information, family characteristics, work situation, social security, and well-being of these three groups. The pre-designed survey content was modified according to the interview results to form a questionnaire.

In addition, the street interview meant that the interviewer selected the respondents in some pre-selected locations according to certain procedures and requirements. After obtaining the consent of interviewees, a brief survey on the spot was conducted according to the questionnaire questions. This survey adopted the street interview method, which is low-cost and can answer questions during the survey process, thereby improving the reliability of the survey results.

### 4.2. Survey Plan

#### 4.2.1. Survey Object

According to the purpose of the survey, the survey objects were food delivery workers, couriers, and online car-hailing drivers in Hangzhou city. The survey unit was every worker in the three groups.

#### 4.2.2. Sampling Method

This survey adopted multi-stage sampling, namely, the sampling method used in each stage was different. Specifically, first-stage sampling units, namely administrative districts, were selected in Hangzhou City. Second-stage units, namely streets, were selected from the selected administrative districts. Finally, survey units were randomly selected in the streets, namely food delivery workers, couriers, and online car-hailing drivers.

In the first stage, the probability proportionate to size sampling (PPS) method was used to select 3 districts. The 13 administrative districts of Hangzhou city were used as the primary sampling units, and the resident population of the districts was used as the basis for measuring the size of the primary units. The total population of Hangzhou was 11,936,010. The sampling interval: 11,936,010/3 = 3,978,670 was obtained by dividing the total population by the number of districts. The value 40,161 was randomly selected in the range of 1 to 3978670 as the initial sampling code. The remaining two sample codes were: 40,161 + 3,978,670 = 4,018,831, 40,161 + 3,978,670×2 = 7,997,510. Finally, the districts containing the sampling codes were Shangcheng District, Binjiang District, and Qiantang District.

The second stage was to sample 4 streets using stratified random sampling. Before sampling streets, streets with a resident population of less than 100,000 were merged with adjacent streets (the one with the least resident population) until all sampling units had a resident population greater than 100,000. After completing merging, a sampling interval of 649,114 and a starting sampling code of 210,200 were obtained by a similar method as above. The remaining sampling codes were: 859,314, 1,508,428, and 2,157,542. The streets that contain the sampling codes were finally obtained: Wangjiang (including Caihe and Sijiqing), Jianqiao, Changhe, and Xiasha. The results are shown in Table 1.

#### 4.2.3. Sampling Capacity

The determination of the sample capacity should take the population size, the variance of the target population, the level of estimation accuracy, and statistical basic data into account. Taking the confidence level 1−*α* as 95%, the α/2 quantile t on the standard normal distribution was 1.96, and the maximum permissible absolute error was *d* = 5%. Due to the unknown overall number of food delivery workers, couriers and online car-hailing drivers in Hangzhou city, it can be regarded as an infinite population. Therefore, the sample size determination formula without correction coefficient was adopted, and the optimal sample size *n*_0_ was obtained as shown in formula (1).

(1) $$n_{0}=\frac{t^{2}p(1-p)}{d^{2}}=\frac{1.96^{2}\times 0.5(1-0.5)}{0.05^{2}}=384.16\approx 385$$

Since this paper adopts multi-stage sampling, the sampling plan was complicated, and it was difficult to calculate the actual deff, it was assumed to be 1.35, and the adjusted sample size *n*_1_ was obtained as shown in formula (2).

(2) $$n_{1}=n_{0}\times deff=385\times 1.35=519.75\approx 520$$

On this basis, assuming that the recovery rate of valid questionnaires was 90%, the final sample size *n*_2_ could be calculated as shown in formula (3).

(3) $$n_{2}=\frac{n_{1}}{P_{valid}}=\frac{520}{0.9}=577.78\approx 578$$

The number of questionnaires in the paper was finally determined to be 578.

#### 4.2.4. Questionnaire Design and Variable Measurement

The questionnaire in this paper is mainly composed of three parts: basic information of respondents, assessment of well-being, and measurement of factors influencing well-being. The complete questionnaire is in the Appendix A. The specific items and symbol representations of the variables can be seen in Table 2.

Firstly, this paper refers to the study of Luo and Lu [29] in the selection of demographic characteristics.

Secondly, we identified a measure of well-being. Given the equivalence between life satisfaction and subjective well-being, and the operability, breadth, and expressiveness of the scale, this paper mainly referred to the SWLS proposed by Diener et al. [30] to assess the well-being of migrant gig workers. This scale measures the dimension of well-being through 5 items and is scored on a 7-point scale, taking into account both subjective and objective aspects of well-being. In addition, considering the conclusions of the literature [31], it is confirmed that the scale in the paper is valid and feasible in the cross-cultural context, and is widely used. Based on this, this paper changed the scoring form of the overall satisfaction scale to a 5-point type, because of the following two points: (1) the research object was time-sensitive; and (2) this conversion had little effect on the analysis of the questionnaire results. The reliability Cronbach’s alpha level of the questionnaire was found to be 0.804.

Thirdly, we identified a measure of occupation satisfaction. Zhao [32] designed the Occupation Satisfaction Scale for ride-hailing drivers. This scale includes 18 items, which are rated using a 5-point Likert scale. Eight items were selected to measure satisfaction with job flexibility, job content, compensation, and platform or company. Additionally, the scale designed by Luo and Lu [29] was referred to when measuring migrant gig workers’ income satisfaction. Thus, there were a total of 11 items to measure occupation satisfaction, using a 5-point Likert scale, with higher scores indicating greater satisfaction. The reliability Cronbach’s alpha level of the questionnaire was found to be 0.892.

Fourth, we identified measures of social interaction and family characteristics. The scale designed by Xiao [33] was referred to, which contained 10 items, including objective support, subjective support, and utilization of social support. The scale had been used in domestic and foreign research. However, it adopted a multiple-choice method and assigned points according to the options, which was too complicated. Considering the purpose of this paper, the items on interpersonal relationships and team activities were extracted and revised to Likert 5-point scoring. In total, the social interaction scale includes 4 measurement items, and the family characteristics scale includes 2 measurement items. Higher scores indicated more social interaction or more satisfaction with the family. The Cronbach’s alpha levels of the scale were found to be 0.696 and 0.786, respectively.

Finally, we identified a measure of social security. Qi [34] designed a questionnaire to obtain the basic information of couriers, their understanding of social security, and their satisfaction with the current social security policy. This paper mainly referred to 9 items of satisfaction measurement, and changed the options into a 5-point Likert scale. A higher score indicated more satisfaction with social security. The Cronbach’s alpha level of the scale was found to be 0.655.

### 4.3. Survey Implementation

After confirming the topic selection, research framework, objectives, and other aspects of the survey, the survey team selected some streets in Shangcheng District, Binjiang District, and Qiantang District based on the multi-stage sampling results and the final required sample capacity. Wangjiang Street, Jianqiao Street, Changhe Street, and Xiasha Street were used as the survey sites from the four districts. Finally, the street interview method was used to distribute the questionnaires on the spots.

### 4.4. Collection and Statistical Analysis of Questionnaires

A total of 600 questionnaires were collected in this paper, and the final valid number of questionnaires was 587, with an effective rate of 97.8%. After the questionnaires were ordered and data entered, statistical modeling was carried out by SPSS25.0 software and AMOS24.0 software. In this paper, the questionnaires were first analyzed by the current situation, and the descriptive analysis method was used to examine the demographic characteristics of the respondents, including the basic information and the social security situation of the respondents. A well-being analysis was then conducted, using an independent sample *t*-test and a one-way ANOVA to analyze the differences in well-being among respondents in variables such as gender, occupation, education, and salary level. Finally, a structural equation model (SEM) was established to analyze the influencing factors of well-being.

A structural equation model was used for establishing, estimating, and testing causal relationship models, which can test the relationship between observed variables and latent variables, internal latent variables, and external latent variables in the model, which is a common method to analyze the relationship among directly observed variables. The estimating parameters of the structural equation model are generally obtained by maximum likelihood estimation (MLE). In this paper, the normality distribution of the model is verified by performing kurtosis and skewness tests on the data. The degree of fit is the most important indicator in the structural equation model, and the higher the model fit, the higher the fit between the theoretical model and the real data.AMOS24.0 uses chi-square as the result of the fit test; generally the chi-square value *p* > 0.05, but chi-square is susceptible to sample size, so in addition to the chi-square statistics, the chi-square degree of freedom ratio (χ^2^/df), approximate root mean square error (RMSEA),goodness of fit index (GFI), modified goodness of fit index (AGFI), comparative fitting index (CFI), value-added fit index (IFI), and Tucker–Lewis index (TLI) were used. In general, models meeting the following criteria were considered well fit: χ^2^/df < 3, RMSEA < 0.08, GFI > 0.90, AGFI > 0.90, CFI > 0.90, IFI > 0.90, and TLI > 0.90. When the actual model is more complex, the model fitting effect can be considered acceptable if the index values are close to the above values.

## 5. Results

### 5.1. Results of Validity and Reliability Test

The validity and reliability of this paper were acceptable and could be used for subsequent factor analysis, which could fulfill the research. The overall Cronbach’s alpha reliability coefficient was 0.911, and each dimension was above 0.7 or close to 0.7, indicating that the sample data were relatively reliable and met the research requirements. The overall Kaiser–Meyer–Olkin (KMO) was 0.894, the KMO of each dimension was above 0.6, and the Bartlett sphericity test was passed (*p* < 0.001).

### 5.2. Results of Current Situation Analysis

#### 5.2.1. Brief Information

In this section, we conducted a descriptive analysis on demographic variables such as gender, occupation, area, age, education, income, health status, and marital status. The results are shown in Table 3.

It can be seen from Table 3 that the distribution of this survey met the requirements of the sampling survey. In general, the gig workers were mainly young and middle-aged men, who were healthy, non-local, married, low educated, and with high-income. Specifically, the gender results show that men accounted for 83.6% and women accounted for 16.4%. Occupation shows that food delivery workers accounted for the highest proportion (33.9%), followed by online car-hailing drivers (33.6%), and finally couriers (32.5%). The area results show that the number of residents outside the Zhejiang province was the largest (63.5%), followed by non-Hangzhou residents (24.5%) and local residents (11.9%). In other words, non-Hangzhou residents accounted for 88%. Therefore, most of the research objects in this paper belong to migrant gig workers. The age results show that most of them were in the age range of 20–39, among which the number of workers aged between 20 and 29 accounted for 44.3%, followed by the workers aged between 30 and 39 (34.4%), and then between 40 and 49 (14%), under 20 (4.1%), and over 50 (3.2%). Education results show that the number of workers with a high school degree accounted for 51.4%, and workers with bachelor’s degree or above only accounted for 4.3%, indicating that the workers received less schooling. Income results show that the income of the workers was generally high, with most of the monthly incomes reaching more than CNY 6000, and only 3% had incomes of CNY 4000 and below. Health results show that most of the workers were in good physical condition, and there was little difference in health before and after engaging in this occupation. The marriage results show that most of the workers were married.

#### 5.2.2. Social Security

**Figure 3:**
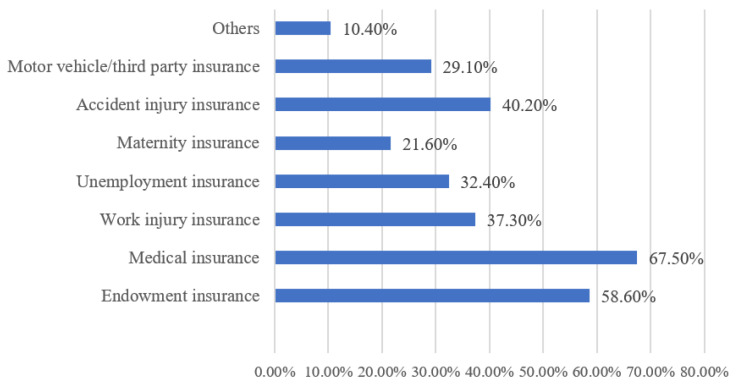
Percentage of Cases with the “Social Security Paying” Option.

The social security of migrant gig workers is closely related to their well-being, and the results are shown in Figure 3. In general, migrant gig workers were most concerned about endowment insurance and medical insurance, and the social insurance payment situation was not ideal. Specifically, the number of people who paid endowment insurance and medical insurance was the largest, accounting for 58.6% and 67.5%, respectively. The remaining insurance contributions were accident injury insurance (40.2%), work injury insurance (37.3%), unemployment insurance (32.4%), motor vehicle/third party insurance (29.1%), and maternity insurance (21.6%).

### 5.3. Normality Test Results

The estimating parameters of the SEM are generally obtained by MLE. Theoretically speaking, MLE requires that the data meet the requirements of a multivariate normal distribution and a sufficiently large sample size. However, in practice, it is difficult to test the assumption of multivariate normal distribution, and researchers generally use a univariate normality test to determine whether the above requirements are met. To this end, this paper conducted kurtosis and skewness tests on the data, and the results show that the skewness value of each variable was between −0.982 and 2.221, and the kurtosis value was between −1.907 and 2.946, which conformed to the normal distribution without exceeding the critical value.

### 5.4. Well-Being Evaluation Results

The previous questionnaire design and variable measurement part combed the influence of family characteristics, occupation satisfaction, social security and social interaction on well-being from the perspective of literature. Since the above five aspects are represented by multiple items, to summarize the multiple items into a whole, this section explores the well-being of migrant gig workers by calculating their average levels. The statistical results are displayed in Table 4.

Table 4 shows that migrant gig workers were satisfied with their family situation and the average score was 3.82. Average scores of social interaction, occupation satisfaction, and well-being were 3.45, 3.07, and 2.95, respectively; there was prominent dissatisfaction with social security with an average score of 2.66. Therefore, the protection of the rights and interests of this group needs to be intensified.

Table 5 and Table 6 show the results of the difference analysis of demographic variables and the post-hoc least significant difference (LSD) test. It can be seen that, at the 5% significance level, the well-being of men was significantly lower than that of women. The well-being of food delivery workers was significantly lower than that of other workers. In addition, the well-being of workers with junior high school education and below was significantly lower than that of people with other degrees. Except for a few extremely low-income people, the well-being of low-income people was lower than that of high-income people, but the difference was not significant.

### 5.5. Results of Analysis about Factors Affecting Well-being

#### 5.5.1. Measurement Model Results

**Figure 4:**
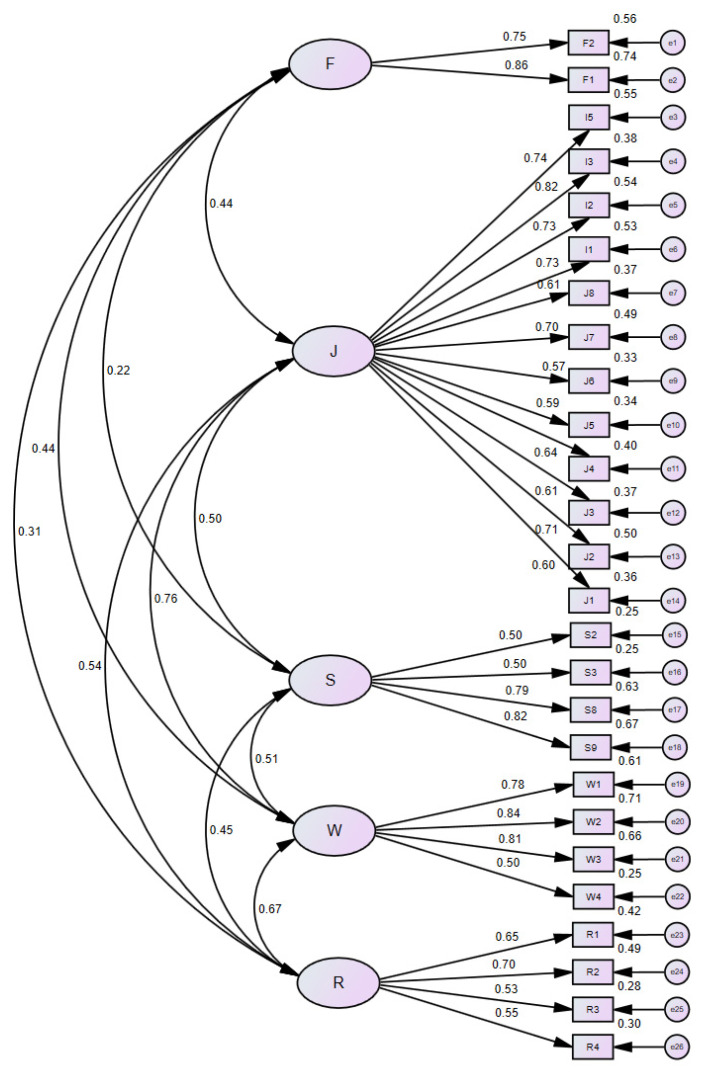
The Optimal Standardized Confirmatory Factor Analysis Model.

Based on the divided dimensions and collected data, combined with the test hypothesis and the initial measurement model verification results, and on the premise of not violating the theory, and the aim to improve the standardized path coefficient and the overall fitting coefficient between the observed variable and the latent variable, the optimal measurement model was obtained as shown in Figure 4.

From the perspective of convergent validity, it can be seen from Table 7 that the average variance extraction (AVE) value of each latent variable was basically above 0.4, the composite reliability (CR) values were all higher than 0.7, and the factor loadings were all higher than 0.5 or close to 0.5. The convergent validity of the model was acceptable.

From the perspective of discriminant validity, $\sqrt{\mathrm{AVE}}$ in Table 8 was basically larger than the correlation coefficient between latent variables, and only a few did not meet the standard, so the model was considered to be within the acceptable range.

From the perspective of structure validity, it can be seen from Table 9 that although $\chi^{2}/df$ exceeded the critical value of 3, the root mean square error of approximation (RMSEA) was less than 0.08, and other indicators were basically greater than 0.8 and close to 0.9. Therefore, the construct validity of the model was acceptable.

In summary, the optimal measurement model contained five latent variables, namely: family characteristics, occupation satisfaction, social security, social interaction, and well-being, including two, twelve, four, four, and four observed variables, respectively. The path coefficients between each latent variable and the observed variable were between 0.495 and 0.862, all of which passed the hypothesis test with a significance level of 0.001. The path coefficients of social interaction and well-being were larger than the $\sqrt{\mathrm{AVE}}$, and others met the requirements. Finally, the overall fitting coefficients of the model also mostly met the requirements. Through the above analysis, the final optimized measurement model here was acceptable, and the confirmatory factor analysis results could meet the research requirements.

#### 5.5.2. Structural Equation Model Results

(1) Results of the Initial Structural Equation Model

The construction of the structural equation model relies on existing theoretical guidance. The hypothesis stated in Section 2 is based on the existing research results. Based on this, family characteristics, social security, occupation satisfaction, social interaction, and well-being were used as latent variables, and 26 items were used as observed variables to construct the initial structural equation model.

After constructing the model, Table 10 indicates that the overall goodness of fit was general, the $\chi^{2}/df$ was 3.665, which was greater than the critical value of 3, the RMSEA value was close to 0.08, and the rest of the indicators were much smaller than 0.9. In addition, the standardized path coefficient between family characteristics and well-being was 0.083, failing the hypothesis test at a significance level of 0.05.

Based on the above analysis, the initial model needed to be revised from two aspects: (1) simplification of the model, i.e., deleting some insignificant path coefficients, so the path between family characteristics and well-being was removed; (2) expansion of the model, i.e., according to the modified index to add some path coefficients. The above corrections needed to be carried out on a theoretical basis, and the specific correction process is shown in Table 11.

The path between family characteristics and well-being was removed because the path coefficient between variables was not significant. In addition, the added error terms were e1⟷e2, e5⟷e7, e7⟷e8, e15⟷e17, e15⟷e18, e17⟷e20, e19⟷e22, e20⟷e21. The correlation between the error terms indicated there was a correlation between the corresponding observed variables. For example, e20⟷e21 corresponded to the sense of occupation achievement and occupation competency. Migrant gig workers who have a sense of accomplishment in their work can often be competent for the occupation, and vice versa.

(2) Results of the Modified Structural Equation Model

**Figure 5:**
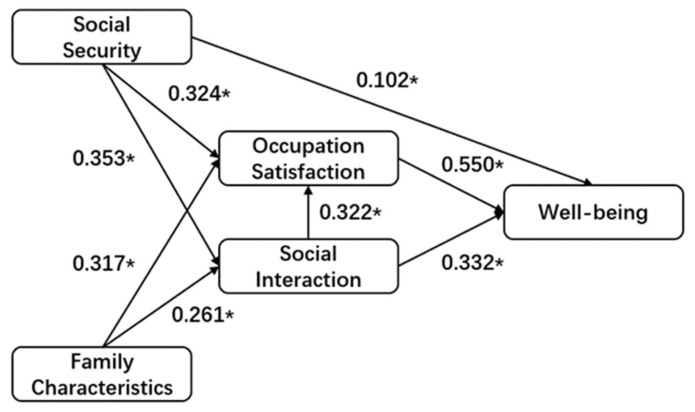
Modified SEM (* *p* < 0.05).

After the above corrections, the structural equation model shown in Figure 5 was obtained. It can be seen from Table 12 that the overall goodness of fit met the requirements, $\chi^{2}/df$ did not exceed the critical value of 3, RMSEA was less than 0.08, and other indicators had been improved and almost reached 0.9. From the results in Table 13, it can be seen that the standardized coefficients of each path had passed the hypothesis test with a significance level of 0.05, the path coefficients range was between 0.102 and 0.550, and the factor loadings were generally positive.

(3) Results of Hypotheses Testing

In addition, it can be seen from Table 13 that occupation satisfaction positively influenced well-being with a prominent effect of when B = 0.550, *p* <0.01, indicating that for every 1 unit increase in occupation satisfaction, migrant gig workers’ well-being would increase by 0.550 units, therefore verifying H2. Social security positively influenced well-being with the prominent effect of when B = 0.102, *p* = 0.036, significance level = 0.05, indicating that for every 1 unit increase in social security, workers’ well-being would increase by 0.102 units, verifying H3a. Social interaction positively influenced well-being, verifying H4 when B = 0.332, *p* < 0.001; for every 1 unit increase in social interaction, workers’ well-being would increase by 0.332 units. Family characteristics positively influenced occupation satisfaction, verifying H1c when B = 0.317, *p* < 0.001; for every 1 unit increase in family characteristics, workers’ occupation satisfaction would increase by 0.317 units. Social security positively influenced occupation satisfaction, verifying H3b when B = 0.322, *p* < 0.001; for every 1 unit increase in social security, workers’ occupation satisfaction would increase by 0.322 units. Family characteristics positively influenced social interaction, verifying H1bwhen B= 0.261, *p* <0.001; for every 1 unit increase in family characteristics, workers’ social interaction would increase by 0.261 units. In addition, during the revision process, the model indicated that social interaction positively influenced occupation satisfaction when B = 0.322, *p* < 0.001, which meant that for every 1 unit increase in social interaction, workers’ occupation satisfaction would increase by 0.322 units. Social security positively influenced social interaction when B = 0.353, *p* < 0.001, which meant that for every 1 unit increase in social interaction, workers’ social interaction would increase by 0.353 units. However, H1ahad not been verified at the significance level of 0.05. Therefore, this paper considers that there is no correlation between the two, and deletes this path.

## 6. Discussion

### 6.1. Results Discussion

In this study, 88% of participants were migrant gig workers. In China, many people choose to work in cities, and the entry threshold for the gig economy is low, so there are many migrant gig workers. At the same time, this study also finds that migrant gig workers have low levels of education, low occupation satisfaction, lack of social security, and low well-being. These results are consistent with existing findings [7,13,14]. Therefore, more attention should be paid to the living conditions among migrant gig workers.

From the perspective of the difference analysis of demographic variables, it could be found that men, food delivery workers, and workers with low education had lower well-being. The above results are consistent with the existing research conclusions [27]. Firstly, compared with women, men have less time to interact with relatives, friends, colleagues, etc., due to the pressure of raising a family. Secondly, with the competition among delivery platforms and the continuous refinement of the system, food delivery workers have a shorter delivery time, a higher probability of complaint than other occupations, and fewer opportunities for appeal, which greatly affects this group’s well-being. Finally, people with higher degrees are less likely to be discriminated against in the workplace and therefore have higher levels of well-being. However, there was no significant difference in well-being among respondents with different incomes, which is inconsistent with the existing studies [22,23]. The reason for this may be that well-being can be influenced by other factors than just income alone [35].

In terms of factors influencing well-being, occupation satisfaction, social interaction, and social security all positively influenced the well-being of migrant gig workers, but family characteristics had no effect. Specifically, occupation satisfaction had the most significant positive impact on well-being, followed by social interaction and social security. In addition, this paper also found that social interaction had a positive effect on occupation satisfaction, and social security had a significant positive effect on social interaction. The specific conclusions are as follows:

(1) The impact of occupation satisfaction on well-being

Occupation satisfaction positively impacted the well-being of migrant gig workers. Among them, income satisfaction had the largest load, indicating that the income satisfaction greatly affected well-being. This conclusion is consistent with previous studies [24]. Ray [36] pointed out that there was a significant positive correlation between occupation satisfaction and well-being. Particularly, job income and quality were very important to workers’ well-being.

(2) The impact of social security on well-being

Social security positively impacted the well-being of migrant gig workers. Social security included social insurance and housing security, enabling individuals to prevent risks and enhance their sense of security, thus positively affecting their well-being. Ewers et al. [37] found that the major determinant of migrant well-being was contract-related matters, including rights, treatment, and medical care. Additionally, the second factor was accommodation and living conditions. This group is often far away from their hometown and often works in outdoor places, facing great uncertainty in safety hazards and accidents. Social security can reduce the risk of uncertainty to a certain extent, thereby enhancing their sense of security and well-being.

(3) The impact of social interaction on well-being

Social interaction positively impacted the well-being of migrant gig workers. This conclusion is consistent with previous studies [24]. Shirmohamm et al. [38] proposed that emotional support from friends and community members was significantly associated with better life satisfaction and less depression and anxiety. Social support obtained from social interaction can also bring individuals a sense of security and stable future expectations, thereby enhancing their sense of well-being.

(4) The impact of family characteristics on occupation satisfaction

Family characteristics positively impacted the occupation satisfaction of migrant gig workers. This result is consistent with the conclusion of Yin [39].The study found that good family relationships could improve occupation satisfaction, while disputes within the family led to lower family satisfaction and lower occupation satisfaction. Workers can improve occupation satisfaction by getting support from family members, and positive experiences at home can improve the mood at work.

(5) The impact of social security on occupation satisfaction

Social security positively impacted the occupation satisfaction of migrant gig workers. This conclusion is consistent with previous studies [23]. Social security often involves a series of livelihood issues, and the sense of security brought by social security can make individuals feel more at ease in their work. Liu and Ding [40] believed that if enterprises could provide social security for residents, such as medical insurance and endowment insurance, it would significantly promote the improvement of their occupation satisfaction. However, due to the particularity of their occupations, migrant gig workers often cannot enjoy some social security due to labor contracts and other issues.

(6) The impact of family characteristics on social interaction

Family characteristics positively impacted the social interaction of migrant gig workers. Family characteristics contain family life and family relationships, and social interaction includes non-family relationships such as colleagues, friends, and neighbors, all of which are social relationships. Family characteristics can influence the establishment of non-family relationships. For example, Zhang et al. [41] believed family troubles would affect an individual’s status at work, including getting along with colleagues.

(7) The impact of social interaction on occupation satisfaction

Social interaction positively impacted the occupation satisfaction of migrant gig workers. Social interaction includes social relationships such as colleagues, friends, and neighbors. Various social relationships constitute a social network. The results of this paper show that the relationship between colleagues had the greatest impact on social interaction. The quality of the relationship between colleagues will affect occupation satisfaction. Brett and Kerk [42] found that the more frequent the interaction between colleagues, the higher the occupation satisfaction.

(8) The impact of social security on social interaction

Social security positively impacted the social interaction of migrant gig workers. Ruan [43] proved that there was a positive relationship between social security support and urban integration. Moderate social security can therefore increase the interaction of migrant gig workers with colleagues, neighbors, or others in their social network.

(9) Family characteristics and well-being

Family characteristics include family life and family relationships, reflecting the satisfaction of family life and the harmony of family relationships. Zhang [44] pointed out that family factors reflecting trust, communication, and tolerance among family members could impact the well-being of Chinese residents. However, the results obtained by the structural equation model in this paper show that family characteristics did not significantly affect the well-being of migrant gig workers due to different studied groups. Results showed that 88% of the people concerned here were from non-Hangzhou areas and were migrant workers. They usually were separated from their families. Thomas et al. [45] noted that the effect of family characterizes on well-being was enhanced by the weakening of other relationships. Conversely, support from family members may be replaced by support from other people. Because of distance, migrant gig workers are more likely to get emotional support from interactions with friends, colleagues, and neighbors than from family members. In addition, Ning [46] found that the well-being of migrant workers was related to family factors, but age difference led to diverse results. For example, migrant workers under 25-years-oldweremore concerned with themselves than their families, so their well-being was irrelevant to family factors. Since nearly 50% of the respondents were between the ages of 20 and 29, it is possible that family characteristics have no significant effect on well-being.

### 6.2. Suggestions

According to the research results, in order to enhance the well-being of migrant gig workers and improve their living conditions, this paper proposes the following policy suggestions:

(1) Optimize the assessment mechanism and promote skill improvement

First of all, companies should re-examine the assessment system for migrant gig workers and avoid assessment based on customer experience only. Specifically, the platform can use historical data to refine the reward and punishment mechanism, such as the introduction of time and space factors, and the establishment of different assessment standards for different periods, densely populated areas, and non-dense areas. In addition, identifying customers with frequent disputes will weaken their influence on the performance evaluation of workers. Secondly, enterprises should strengthen the professional skills of migrant gig workers and establish a complete career promotion channel. Finally, relevant government departments can cooperate with enterprises to conduct skill appraisals and publicly commend high-skilled persons.

(2) Step up the pilot construction of rights protection and strengthen the supervision of enterprises

On the one hand, the government should optimize the labor rights protection system for migrant gig workers including labor relationship identification, working hour definition, and social insurance protection, accelerate the pilot construction, and summarize the successful experience and mature practices promptly. In addition, during pilot construction, the government should intensify the supervision to prevent enterprises from using various means to shirk their responsibilities. For example, food delivery platforms use outsourcing companies to avoid signing labor contracts and even allow food delivery workers to sign documents such as an automatic waiver of social security payments. On the other hand, the government should relax the application threshold for applying for housing security. Most of the migrant gig workers are from non-Hangzhou areas, and housing is a big problem. The government’s current rental policy has limitations on education, labor contracts, and social security payments. Most of the survey respondents in this paper failed to meet the above conditions and could not enjoy housing subsidies, which increases the pressure of living to a certain extent.

(3) Bring the cohesive role of the community and trade unions into full play

First of all, the community should provide opportunities and platforms for cultural exchange and improvement with urban residents, enhance the cultural identity and integration between the two, and narrow the social distance. Secondly, the government should introduce relevant policies in combination with skill improvement, and promote the education of migrant gig workers according to skill rating. For instance, tuition reduction and exemption, and necessary education improvement consultation could be provided for high-skilled migrant gig workers. Finally, the trade union should increase the frequency of festival activities, increase the participation of migrant gig workers, and enrich their spiritual life.

### 6.3. Limitations

This paper has the following shortcomings, which need further study:

Sample limitations. Firstly, the research objectives are limited to migrant gig workers in Hangzhou city, represented by food delivery workers, couriers, and online car-hailing drivers. Secondly, the research scope is limited to Hangzhou city. Thirdly, some similar occupations are not involved (for instance, Internet housekeeping services). In further research, the research scope should be expanded and regional restrictions should be removed [47].

Factor limitations. This paper only selects a few key factors affecting well-being. In further research, other related variables, such as personality traits [48], should be taken into account.

## 7. Conclusions

By issuing questionnaires, this paper evaluated the well-being of couriers, food delivery workers, and online car-hailing drivers in Hangzhou, and analyzed the factors affecting well-being. The results show that migrant gig workers were indispensable in Hangzhou city. They had distinct personal characteristics, and their overall well-being was low due to various factors. There were problems such as low occupation satisfaction and lack of social security. Specifically:

(1) Migrant gig workers had distinct characteristics, low well-being, and were affected by various factors

With distinct characteristics, the migrant gig workers were mainly middle-aged migrant men, with low educational backgrounds and separation from families. The well-being of this group was low, and there were significant differences in demographic variables such as gender, education, and occupation. The well-being of men, those with low-education, and food delivery workers was significantly lower than that of others. Well-being was affected by occupation satisfaction, social interaction, and social security, in turn.

(2) Migrant gig workers encountered low occupation satisfaction, high influence of income, and limited occupation development

As the most important factor affecting well-being, the occupation satisfaction of migrant gig workers was low. However, income satisfaction and return fairness were the most significant factors affecting occupation satisfaction. These two factors are closely related to the salary system. Although the income of this group is relatively considerable, the entry threshold for this occupation is low, and workers are short of professional skills. They can only rely on high-intensity work to gain advantages.

(3) Migrant gig workers faced the lack of social security, great impact of housing insurance, and insufficient social insurance coverage

As a significant factor affecting well-being, the social security satisfaction of migrant gig workers was low. Housing security was the most vital factor in measuring social security. In addition, there were problems such as a low social insurance participation rate and limited types of insurance.

The results of this paper have both theoretical and practical significance. From the theoretical point of view, this paper enriches the research on the living conditions of migrant gig workers. Previous studies usually only focus on migrant gig workers in a broad sense or a specific occupation, ignoring the differences and similarities among different gig jobs. In this paper, migrant gig workers with a similar nature and labor force composition are studied together, and the largest number of food delivery workers, couriers, and online car-hailing drivers are taken as representatives of migrant gig workers to explore their living conditions, which can fulfill this research gap. Moreover, the previous literature rarely explored the evaluation of migrant gig workers on their own lives. This paper uses well-being as an indicator of life evaluation, confirms the previous literature on the influence of occupation satisfaction, social interaction, and social security on well-being, and explores the relationship between these factors, enriching the research on factors affecting well-being. From the practical point of view, suggestions from the perspective of optimizing the mechanism, pilot construction, and platform provision [49] can further promote the healthy development of the gig economy, narrow the gap between the rich and the poor, and promote social equity.

## Figures and Tables

**Table 1:** Street Sampling Results.

District	Street	Resident Population	Meaged Street	Resident Population	Sampling Code
Shangcheng District	Qingbo	26,950	Hubin	125,431	
	Hubin	23,385			
	Xiaoying	75,096			
	Wangjiang	85,612	Wangjiang	248,752	210,200
	Caihe	69,602			
	Sijiqing	93,538			
	Nanxing	52,528	Ziyang	146,596	
	Ziyang	94,068			
	Kaixuan	71,747	Zhalongkou	165,905	
	Zhalongkou	94,158			
	Pengbu	114,237	Pengbu	114,237	
	Jianqiao	155,866	Jianqiao	155,866	859,314
	Dinglan	172,753	Dinglan	172,753	
	Jiubao	193,909	Jiubao	193,909	
Bingjiang District	Xixing	143,318	Xixing	143,318	
	Changhe	168,276	Changhe	168,276	1,508,428
	Puyan	192,265	Puyan	192,265	
Qiantang District	Xiasha	335,634	Xiasha	335,634	2,157,542
	Baiyang	186,343	Baiyang	186,343	
	Others	247,173	Others	247,173	

**Table 2:** Measurement of Variables.

Latent Variable	Observed Variable	Items	Symbolic Representation of Observed Variable
Family characteristics (F)	Family life satisfaction	You are satisfied with your family life.	F1
	Family support	Your family members are very supportive and take care of you.	F2
Occupation satisfaction (J)	Flexible working hours	Your working hours are flexible and free.	J1
	Job achievement	This job gives you a sense of accomplishment.	J2
	Job competency	This job brings your expertise to bear, and you are well qualified.	J3
	Work–life balance	This job allows you to achieve a work–life balance and take care of your family.	J4
	Task assignment	The platform/company’s order distribution system (task allocation) is reasonable.	J5
	The company is responsible	The platform/company is responsible for accidents such as traffic.	J6
	Company offers opportunities	The platform/company provides the opportunity to find more employment options, which will benefit your future employment development.	J7
	Satisfaction with the company’s reward and punishment system	You are satisfied with the platform/company’s rewards and punishments.	J8
	Income fairness	You are being fairly rewarded for this work.	I1
	Income satisfaction	You are satisfied with the salary of the job.	I2
	The income can meet the needs of living in Hangzhou	Your current income can meet your living needs in Hangzhou.	I3
	The proportion of consumption in Hangzhou	In addition to general living expenses, your remaining income or savings are mainly spent locally.	I4
	Income status	Your economic income level is in the local middle level.	I5
Social security (S)	Low social security premiums	You think the social security premiums are low.	S1
	High social security benefits	You think social security pays well.	S2
	Social security provides protection	You think social security provides you with protection.	S3
	No social security benefits	You have not received the benefits of social security.	S4
	Social security publicity	You think the propaganda effect of social insurance is not good, a lot of people are not clear.	S5
	Labor contract signing	You have signed a labor contract with the platform/company.	S6
	Attitude towards signing a labor contract	You think it is very necessary to sign a labor contract.	S7
	Knowledge of housing subsidies	You know the housing subsidy in Hangzhou very well.	S8
	Received rousing allowance	You have received the housing subsidy of Hangzhou.	S9
Social interaction (R)	Communicate with friends	You often talk to your friends when you are in trouble.	R1
	Good relationship with colleagues	You have a close and harmonious relationship with your colleagues.	R2
	Good relationship with neighbors	You have a close and harmonious relationship with your neighbors.	R3
	Willing to participate in team activities	You are willing to participate in team activities regularly.	R4
Well-being (W)	Life matches expectations	In most ways your life is close to your ideal.	W1
	Good living conditions	The conditions of your life are excellent.	W2
	Life satisfaction	You are satisfied with your life.	W3
	Got what you want most in your life	So far you have gotten the important things you want in life.	W4
	Unwilling to change your life in the future	If you could start your life over, you would change almost nothing.	W5

**Table 3:** Frequency of Basic Information of Respondents.

Statistical Variables	Category	Percentage/%
Gender	Men	83.6
	Women	16.4
Occupation	Food delivery workers	33.9
	Couriers	32.5
	Online car-hailing drivers	33.6
Area	Hangzhou	11.9
	Zhejiang Province (except Hangzhou)	24.5
	Outside the Zhejiang Province	63.5
Age	Under 20	4.1
	Between 20 and 29	44.3
	Between 30 and 39	34.4
	Between 40 and 49	14.0
	Over 50	3.2
Education	Junior high school degree and below	26.1
	High school degree	51.4
	Junior college degree	18.2
	Bachelor’s degree and above	4.3
Income	Less than CNY 4000	2.9
	CNY 4001–6000	20.6
	CNY 6001–8000	40.9
	More than CNY 8000	35.6
Health status	Excellent	11.2
	Very Good	17.2
	Good	41.7
	Average	28.8
	Poor	1.0
Current health status compared to previous	Much better	6.8
	Better	7.7
	About the Same	59.5
	Worse	22.8
	Much Worse	3.2
Marital status	Unmarried	28.1
	Married	70.7
	Widowed	0.5
	Divorced	0.7

**Table 4:** Statistical Results of Migrant Gig Workers on Various Factors.

Factors	Number of Cases(N)	Minimum (M)	Maximum (X)	Mean (E)	Standard Deviation (SD)
Family characteristics(F)	587	1	5	3.8203	0.64829
Occupation Satisfaction(J)	587	1.92	5	3.0726	0.59111
Social Security(S)	587	1	5	2.6567	0.65887
Social Interaction(R)	587	1.75	5	3.4505	0.57354
Well-being(W)	587	1	5	2.9522	0.69549

**Table 5:** Differences in Gender on Various Factors.

Factors	Men (N = 361)	Women (N = 73)	T-Value	*p*-Value
F	3.041 ± 0.585	3.228 ± 0.599	−2.483	0.013
J	3.841 ± 0.627	3.719 ± 0.741	1.463	0.144
S	2.627 ± 0.642	2.805 ± 0.722	−2.114	0.035
R	3.431 ± 0.558	3.548 ± 0.64	−1.595	0.111
W	2.903 ± 0.693	3.195 ± 0.659	−3.311	0.001

**Table 6:** Differences in Well-being on Various Factors.

Genre		Well-Being		F-Value	LSD
		E	SD		
Occupations	Food delivery workers	2.708	0.698	24.3 *	1 < 2,3
	Couriers	2.872	0.626		
	Online car-hailing drivers	3.236	0.66		
Education	Junior high school and below	2.833	0.657	4.164 *	4 < 5,6,7
	High school	2.965	0.611		
	Junior college	2.971	0.806		
	Bachelor’s and above	3.413	1.033		
Income	Less than 4000	3.231	0.78	2.33	
	4001–6000	2.831	0.759		
	6001–8000	2.941	0.669		
	More than 8000	3.028	0.663		

Note: * represents *p* < 0.05, 1 represents food delivery workers, 2 represents couriers, 3 represents online car-hailing drivers,4 represents junior high school and below, 5 represents high school, 6 represents junior college, and 7 represents bachelor’s and above.

**Table 7:** Normalized Path Coefficients of Optimal Measurement Models.

Path			Estimate	*p*-Value	AVE	CR
F2	<---	F	0.751		0.654	0.790
F1	<---	F	0.862	***		
J8	<---	J	0.612		0.430	0.900
J7	<---	J	0.700	***		
J6	<---	J	0.573	***		
J5	<---	J	0.587	***		
J4	<---	J	0.635	***		
J3	<---	J	0.606	***		
J2	<---	J	0.705	***		
J1	<---	J	0.597	***		
I1	<---	J	0.727	***		
I2	<---	J	0.735	***		
I3	<---	J	0.617	***		
I5	<---	J	0.739	***		
S2	<---	S	0.497		0.447	0.754
S3	<---	S	0.495	***		
S8	<---	S	0.793	***		
S9	<---	S	0.816	***		
R1	<---	R	0.647		0.373	0.701
R2	<---	R	0.702	***		
R3	<---	R	0.528	***		
R4	<---	R	0.548	***		
W1	<---	W	0.779		0.555	0.828
W2	<---	W	0.841	***		
W3	<---	W	0.810	***		
W4	<---	W	0.498	***		

Note: *** denotes *p* < 0.001. The blank space in the table means that the path coefficient was set to 1, so there is no *p*-value.

**Table 8:** Table of Normalized Path Coefficients Between Optimal Measurement Model Factors.

	F	J	S	R	W
F	0.654				
J	0.438 ***	0.430			
S	0.225 ***	0.503 ***	0.447		
R	0.305 ***	0.536 ***	0.448 ***	0.373	
W	0.442 ***	0.764 ***	0.514 ***	0.673 ***	0.555
$\sqrt{\mathrm{AVE}}$	0.809	0.656	0.668	0.610	0.745

Note: *** denotes *p* < 0.001.

**Table 9:** Table of Optimal Measurement Model Overall Fitting Coefficient.

$\chi^{2}/df$	RMSEA	GFI	AGFI	CFI	IFI	TLI
3.391	0.074	0.832	0.796	0857	0.858	0.839

**Table 10:** Overall Fitting Coefficient of Initial SEM.

$\chi^{2}/df$	RMSEA	GFI	AGFI	CFI	IFI	TLI
3.665	0.078	0.824	0.788	0.839	0.840	0.820

**Table 11:** Model Correction Process.

Correction Method	Specific Correction Process
Removing path	F→W
Adding error term	e1⟷e2, e5⟷e7, e7⟷e8, e15⟷e17, e15⟷e18, e17⟷e20, e19⟷e22, e20⟷e21

**Table 12:** Overall Fitting Coefficient Table of Modified SEM.

$\chi^{2}/df$	RMSEA	GFI	AGFI	CFI	IFI	TLI
2.629	0.061	0.879	0.849	0.905	0.906	0.890

**Table 13:** Normalized Path Coefficients of Modified SEM.

Path			Estimate	S.E.	C.R.	*p*-Value
R	<---	F	0.261	0.056	4.165	***
R	<---	S	0.353	0.101	5.023	***
J	<---	F	0.317	0.062	5.566	***
J	<---	R	0.322	0.077	5.116	***
J	<---	S	0.324	0.111	5.142	***
W	<---	S	0.102	0.061	2.099	0.036
W	<---	J	0.550	0.053	7.316	***
W	<---	R	0.332	0.056	5.114	***
S2	<---	S	0.465			
S3	<---	S	0.458	0.133	7.900	***
S8	<---	S	0.798	0.242	8.857	***
S9	<---	S	0.833	0.224	8.850	***
R1	<---	R	0.709			
R2	<---	R	0.719	0.086	10.567	***
R3	<---	R	0.512	0.110	7.605	***
R4	<---	R	0.457	0.097	7.720	***
F1	<---	F	0.816			
F2	<---	F	0.793	0.114	8.361	***
I5	<---	J	0.730			
I3	<---	J	0.617	0.053	12.361	***
I2	<---	J	0.731	0.063	14.702	***
I1	<---	J	0.722	0.060	14.514	***
J8	<---	J	0.564	0.067	11.208	***
J7	<---	J	0.676	0.074	13.565	***
J6	<---	J	0.544	0.067	10.812	***
J5	<---	J	0.544	0.066	10.847	***
J4	<---	J	0.623	0.069	12.450	***
J3	<---	J	0.601	0.063	11.969	***
J2	<---	J	0.693	0.071	13.879	***
J1	<---	J	0.588	0.066	11.737	***
W4	<---	W	0.489			
W3	<---	W	0.805	0.148	10.013	***
W2	<---	W	0.832	0.156	10.129	***
W1	<---	W	0.778	0.134	9.881	***

Note: *** denotes *p* < 0.001.

## Data Availability

The data used to support the findings of this study are available from the first author upon request.

## Appendix A

Questionnaire on Well-being of Migrant Gig Workers in Hangzhou

Dear Sir/Madam,

Hello! Thank you for taking time out of your busy schedule to accept this survey. The survey aims to understand the well-being of gig workers in the main urban areas of Hangzhou. This survey is conducted in an anonymous way, and the survey results are for academic research only. Personal information involved will be kept strictly confidential, so you are expected to answer according to the actual situation. Thank you very much for your understanding and cooperation. Wish you a happy life!

Part I Individual Characteristics

1. Your gender is_____.

A. Male B. Female

2. What is your occupation?

A. Delivery worker B. Courier C. Online car-hailing driver

3. You are from_____.

A. Hangzhou B. Zhejiang Province(except Hangzhou)

C. Outside the Zhejiang Province

4. Your current age is_____.

A. Under 20 B. Between 20 and 29 C. Between 30 and 39 D. Between 40 and 49 E. Over 50

5. Your educational background is_____.

A. Junior high school degree and below B. High school degree

C. Junior college degree D. Bachelor degree and above

6. What is your average monthly salary?

A. Less than 4000 YuanB.4001–6000YuanC.6001–8000Yuan

D. More than 8000 Yuan

7. Overall, how do you fell about your health status?

A. Excellent B. Very good C. Good D. Average E. Poor

8. Compared with before you took this job, how do you feel about your current health status?

A. Much better B. Better C. About the Same D. Worse E. Much worse

Part II Family Characteristics

9. What is your marital status?

A. Unmarried B. Married C. Widowed D. Divorced

10. Where do your children go to school?

A. No children or children not attending school B. Children go to school in their hometown C. Children go to school in Hangzhou

11. The type of school your child attends is____. (Omitted if A or B was selected in question 10)

A. Public school B. Private school

12. Please read the following items carefully, select the answer that is most consistent with your actual situation, and tick the corresponding boxes.1 = strongly disagree, 5 = strongly agree.

Item description\Options	1	2	3	4	5
You are satisfied with the school your child attends.					
You are satisfied with your family life.					
Your family members are very supportive and take care of you.					

Part III Work situation and economic factors

13.Please read the following items carefully, select the answer that is most consistent with your actual situation, and tick the corresponding boxes. 1 = strongly disagree, 5 = strongly agree.

Item description\Options	1	2	3	4	5
Your working hours are flexible and free.					
This job gives you a sense of accomplishment.					
This job brings your expertise to bear, and you are well qualified.					
This job allows you to achieve a work–life balance and take care of your family.					
You are being fairly rewarded for this work.					
You are satisfied with the salary of the job.					
The platform/company’s order distribution system (task allocation) is reasonable.					
The platform/company is responsible for accidents such as traffic.					
The platform/company provides the opportunity to find more employment options, which will benefit your future employment development.					
You are satisfied with the platform/company’s rewards and punishments					
Your current income can meet your living needs in Hangzhou.					
In addition to general living expenses, your remaining income or savings are mainly spent locally.					
Your economic income level is in the local middle level.					

Part IV Social security and Social interaction

14. What insurance have you paid for?

A. Endowment insurance B. Medical insurance C. Work injury insurance

D. Unemployment insurance E. Maternity insurance

F. Accident injury insurance G. Motor vehicle/third party insurance H. Others

15.Please read the following items carefully, select the answer that is most consistent with your actual situation, and tick the corresponding boxes. 1 = strongly disagree, 5 = strongly agree.

Item description\Options	1	2	3	4	5
You think the social security premiums are low.					
You think social security pays well.					
You think social security provides you with protection.					
You have not received the benefits of social security.					
You think the propaganda effect of social insurance is not good, a lot of people are not clear.					
You have signed a labor contract with the platform/company.					
You think it is very necessary to sign a labor contract.					
You know the housing subsidy in Hangzhou very well.					
You have received the housing subsidy of Hangzhou.					
You often talk to your friends when you are in trouble.					
You have a close and harmonious relationship with your colleagues.					
You have a close and harmonious relationship with your neighbors					
You are willing to participate in team activities regularly.					

Part V Well-being

16.Please read the following items carefully, select the answer that is most consistent with your actual situation, and tick the corresponding boxes. 1 = strongly disagree, 5 = strongly agree.

Item description\Options	1	2	3	4	5
In most ways your life is close to your ideal.					
The conditions of your life are excellent.					
You are satisfied with your life.					
So far you have gotten the important things you want in life.					
If you could live your life over, you would change almost nothing.					

This questionnaire survey is over. Thank you for your understanding and cooperation. Wish you a happy life again!
